# Validation of a Visually Aided Dietary Assessment Tool to Estimate Dietary Intake in an Adult Swiss Population

**DOI:** 10.3389/fnut.2022.844156

**Published:** 2022-04-28

**Authors:** Gilles Nève, Laura Bur, Ladina Lampert, Christoph Höchsmann, Christine Brombach, Nina Steinemann, Arno Schmidt-Trucksäss

**Affiliations:** ^1^Division of Sports and Exercise Medicine, Department of Sport, Exercise and Health, University of Basel, Basel, Switzerland; ^2^Pennington Biomedical Research Center, Baton Rouge, LA, United States; ^3^Department of Sport and Health Sciences, Technical University of Munich, Munich, Germany; ^4^Institute of Food and Beverage Innovation, Zurich University of Applied Sciences, Life Sciences and Facility Management, Wädenswil, Switzerland; ^5^Department of Epidemiology, Institute for Epidemiology, Biostatistics and Prevention, University of Zurich, Zurich, Switzerland

**Keywords:** diet assessment tool, weighed food record, dietary intake, validation study, food frequency questionnaire

## Abstract

**Background:**

Accurately assessing dietary intake is crucial for understanding how diet affects a person’s health. In large cohorts, paper-based dietary assessment tools (DAT) such as food recalls or food frequency questionnaires have emerged as valid tools with a low burden for participants.

**Objective:**

To validate a visually aided DAT for use in studies with Swiss adults against the gold standard of a weighed 7-day food record (7 d-FR).

**Design:**

Fifty-one adults (*n* = 24 women, *n* = 27 males) participated in the study and were recruited within two age groups (20–40 and 50–70 y). Each participant filled out the visually aided DAT, then the 7 d-FR. The DAT was compared to the 7 d-FR for total energy intake, macronutrients, sugar, water, and portions of fruits and vegetables. Pearson correlation and Bland–Altman analyses were used for statistical analyses.

**Results:**

Total correlations ranged from 0.288 (sugar, *p* < 0.05) to 0.729 (water, *p* < 0.01). The older age group showed higher correlations for total energy intake, protein, fats, carbohydrates, and sugar, but not for water (*p* < 0.05). Correlations were moderate at *r* > 0.5, whereas only water and protein reached those values in the young group. Both groups overestimated total calories in kcal (+14.0%), grams of protein (+ 44.6%), fats (+36.3%), and portions of fruits and vegetables (+16.0%) but strongly underestimated sugar intake (−50.9%).

**Conclusion:**

This DAT showed that all macronutrients and total energy intake were estimated more accurately by the older age group and therefore might be adequate to capture dietary habits in older Swiss adults.

## Introduction

The assessment of dietary intake in adults is one of the key elements in risk stratification when assessing chronic diseases such as diabetes, cardiovascular diseases, or other lifestyle-related non-communicable diseases (1). Until now, there has been a trade-off between the accuracy of a dietary assessment method, its practicality, and its manageability in clinical trials (2). As such, in scientific settings, 24 h recalls and weighed food records (FR) as retrospective methods are most widely used (3). 24 h recalls aim to assess the food intake consumed in the 24 h before the assessment day. The main advantages and disadvantages of 24 h recalls have been well described (4). FR are most used during a 4- or 7-day period (4 d-FR/7 d-FR), where participants are asked to weigh and (or) report any food/drink item consumed during that time frame. This yields a precise overview of a person’s food consumption, that may give information on dietary patterns during the week and (or) on weekend days. This prospective method, if properly conducted, counteracts a memory bias, which might occur in retrospective methods. However, FRs involve high effort by participants and evaluation of data by the study personnel can be burdensome because of high data volumes (5). Although the FR is considered the gold standard for dietary assessment, several disadvantages should be considered that have been described previously (6, 7). As a possible solution, food frequency questionnaires (FFQ) have emerged as more suitable options for large studies, making up for their lack of precision with their ease of use, the low burden for participants and study personnel, as well as the reduced costs when compared to other methods (3). Further, FFQs assess dietary habits retrospectively, meaning that these habits are not altered during the assessment period (8). Still, retrospectively assessing food intake may affect accuracy, and problems of underreporting or false reporting are recognized. In addition, each FFQ needs to be validated against the gold standard to ensure quality.

It has been extensively studied that study participants tend to answer questionnaires to fit social desirability (9–12). This means that behavioral patterns that are commonly seen as “good” or “healthy” (e.g., daily physical activity, being non-smoker) are overreported, whereas patterns and behaviors that are seen as “unhealthy” tend to be underreported (e.g., high consumption of sugary drinks).

In epidemiological studies, it is important to accurately capture nutritional habits such as daily sugar intake or fruit and vegetable consumption. The amount of fruits and vegetables consumed is important since an inverse association between fruit and especially vegetable consumption and the prevalence of metabolic syndrome has been reported in a meta-analysis (13–15). The World Health Organization (WHO) recommends a daily intake of five portions of fruits and vegetables or roughly 400 grams for adults (16). Conversely, the WHO recommends that sugar intake should be reduced to a maximum of 50 grams per day, as an increased intake of sugar is directly associated with the risk of obesity (13, 17). To date, one Swiss study has investigated the amount of total sugar intake (in adults) and found that participants consumed 107 grams of sugar per day on average (18). Although that study showed that sugar intake in Switzerland, as assessed *via* 24 h-recall, is lower than in other countries (e.g., Netherlands), it remains more than twice as high as the daily recommendations and Switzerland is listed as one of the European nations with the highest sugar consumption per capita (19, 20).

It was demonstrated that in many international studies, dietary habits (e.g., meal frequency, portion size, number of meals per day) differ greatly between younger and older adults (21–23). At the time of the present study, most validation studies of dietary assessment methods had been conducted in younger adults while the assessment tools are widely used in studies of elderly people. Such tools generally have moderate correlations (*r*-value 0.40–0.59) between the gold standard and the validated assessment tool. Other tools that have been developed specifically for older populations might not be suitable for younger participants (23–25).

The present validation study was part of the Cardiopulmonary Exercise Testing (COmPLETE) study, which tested over 600 healthy adults and 80 patients with heart failure (26).

In the present study, we validated a visually aided dietary assessment tool (DAT) against the gold standard, the 7 d-FR (27). Total energy intake (kilocalories, kcal), macronutrients (grams), as well as water (liters), fruits and vegetables (portions), and sugar intake (grams) were examined. We aimed to assess whether this tool is useful to provide a valid estimate of all macronutrients, as well as for fruits and vegetables, and daily sugar intake. Finally, the present study aims at validating the assessment tool for younger adults, as well as older adults equally.

## Materials and Methods

### Study Population and Design

Study participants were recruited between March and May 2021 through advertisements *via* email, online flyers, and word of mouth in northwestern Switzerland. Eligible participants were 20–40 or 50–70 years of age (sampling stratified by age), and mentally and physically able to follow the study protocol. Exclusion criteria were as follow: illness during the study period that affected diet, substantial lifestyle changes during the study (e.g., smoking cessation, diets), and a cardiac pacemaker since the conducted study included bio-impedance measures for body composition between the first and second visit. Information about present chronic diseases (e.g., heart failure, cancer, diabetes) and the use of medication were collected *via* a telephone interview before the first visit. Additionally, smoking status was assessed before the start of the study. Participants received written information detailing the procedures of the study and they gave written informed consent before participation.

On the first visit, anthropometric measurements were taken, including body composition using the bio-impedance (InBody 720, InBody Co., Ltd., Seoul, South Korea). Then, blood pressure was measured twice after 10 min of rest with an automatic blood pressure monitor system (Omron Healthcare, Germany). Participants were asked to fill out the paper form DAT before they were instructed on how to complete the 7 d-FR. In addition, participants were asked not to change their dietary or physical activity habits during the monitoring period. The second visit occurred 7 or 8 days after the first visit and was identical to it. During the second visit, study personnel verfied that the study protocol was followed and discussed the results of the 7 d-FR with the participants. The sample size for the present study was determined according to a similar validation study from Switzerland (28). The present study was approved by the Ethics Committee of Northwestern and Central Switzerland (EKNZ 2021-00406) and complied with the declaration of Helsinki.

### Dietary Assessment

#### Dietary Assessment Tool

During the first and second visit, to assess habitual food consumption, participants reported which food items they consumed on a “typical day” using the DAT. A “typical day” was defined by the study personnel as a day, on which participants followed a routine they would follow on most weekdays (e.g., normal workday, illness-free). The DAT as used in the present study is provided in the Supplementary Figure. The DAT shows the food pyramid of the Swiss Society for Nutrition (SGE) (Version 2005 – 2011) on the left third of the page, a portion size equivalent for various food items of the respective category in the middle, as well as five mealtimes (breakfast, snack #1, lunch, snack #2, dinner) and a column for the sum of the five mealtimes. The food pyramid is divided into six levels, with several sub-levels, which are as follows:

1st section (top of the pyramid): Sweets (e.g., chocolate, cake, sweet beverages).

2nd section: *part 1*: vegetable oils, butter, nuts.

*Part 2*: fatty meals (e.g., sausages, fried food, cream sauces).

3rd section: *part 1*: Meats and meat-like products (e.g., chicken, fish, tofu, eggs).

*Part 2*: Dairy products (e.g., milk, yogurt, cheese).

4th section: Grains and legumes (e.g., bread, corn flakes, potatoes, pasta, lentils).

5th section: Vegetables and fruits, including fruit juices.

6th section (base of the pyramid): Unsweetened drinks (e.g., water, tea, coffee).

Underneath the pyramid: alcoholic beverages (e.g., beer, wine).

#### Seven-Day Food Record

Between the two visits (7–8 days apart), all participants were instructed to record their dietary intake over seven consecutive days. We used a modified version of the previously validated Freiburg Diet Protocol (29), which was developed by the German Federal Research Institute for Nutrition and Food. The FR was handed out in paper form. Participants were instructed to always keep the FR with them and to fill it out after each food or beverage consumption, irrespective of whether it was a meal or snack, to avoid lack of reporting. All participants received verbal and written instruction on how to keep track of their dietary intake and on how to use the DAT. Each page of the FR included additional written instructions. The FR had pre-defined food categories (e.g., bread, dairy products, legumes), with examples of foods for each category. The categories of the DAT and FR were similar but the FR had more subcategories and food items. Additional space was provided on the paper forms to allow recording of consumed foods not listed. All items were listed with the standard portion sizes, and participants were asked to report the number and size of portions consumed throughout the day or the amount (in grams or milliliters). For best precision, participants were asked to weigh all consumed food items using their own kitchen scale. Because of the COVID-19 pandemic restrictions during the study, restaurants, canteens, and bars were closed, and private gatherings were limited to five people, meaning that most – if not all – meals were consumed at home. This potentially positively affected the precision of the measurements since all participants were asked to weigh the food items with their own kitchen scale. Participants returned the completed 7 d-FR at the time of the second visit and they were able to discuss any issues they had encountered with the FR with the study personnel at that time.

All food items were entered into NutriGuide^®^ Swiss (Version 4.9), an online software solution that calculates nutritional values of single food items, as well as for meals.

### Statistical Analysis

After completion of data collection, all 7 d-FR were checked for plausibility and completeness by the study personnel.

Based on the food groups illustrated in the food pyramid of the DAT, all food items were categorized into sweets, fatty meals, fats, meat/meat-like products, dairy, grains, legumes, drinks, and alcohol. Each food group of the 7 d-FR was matched with the above-listed food group of the DAT. For the DAT, nutritional values in kcal of the portion size equivalents were calculated with NutriGuide^®^ Swiss and multiplied with the number of portions consumed by the participant for each of the above-mentioned food groups. The total was calculated by summing all food groups. For the 7 d-FR, study personnel entered all food items into the NutriGuide^®^ Swiss software, and nutritional values were calculated by averaging the caloric intake of the 7 days recorded.

Prior to data analysis, we tested for normal distribution of the data using the Shapiro–Wilk test and found that the data of both the DAT and 7 d-FR was positively skewed (*p* = 0.01 and *p* = 0.04, respectively) (30). The logarithmic transformation of the data showed no proportional bias (unstandardized β-coefficient = 0.106, *p* = 0.515). Therefore, all data are presented as median and interquartile range (Table 1). To calculate the differences between the medians reported in Table 1, we performed a quantile regression for unpaired samples.

**TABLE 1:** Energy intake by macronutrients and group.

Category		Total	20–40 years	50–70 years
			*n* = 27	*n* = 24
Energy (kcal)	DAT	2171 (1813–2514)	2253 (1955–3011)	1966 (1567–2431)**
	7 d-FR	1934 (1554–2241)	2122 (1702–2769)	1609 (1420–2158)**
	Difference (%)	12.3	6.2	22.2**
Protein (g)	DAT	111.5 (87.5–133.0)	118.2 (88.4–135.3)	109.0 (79.4–123.7)
	7 d-FR	76.4 (59.9–90.3)	80.2 (63.7–97.5)	70.6 (58.6–83.7)
	Difference (%)	45.9	47.4	54.4
Carbohydrates (g)	DAT	183.8 (136.8–248.2)	221.9 (179.3–278.8)	169.7 (111.3–207.6)**
	7 d-FR	206.5 (162.2–265.9)	241.2 (184.0–292.7)	184.6 (137.1–241.9)**
	Difference (%)	−11.0	−8.0	−8.1
Fats (g)	DAT	103.4 (89.2–126.3)	104.9 (90.4–134.8)	91.9 (87.0–122.9)
	7 d-FR	70.6 (58.0–96.1)	76.6 (59.6–115.3)	65.1 (53.1–79.7)
	Difference (%)	46.5	36.9	41.2
Sugar (g)	DAT	39.2 (31.1–54.2)	39.2 (34.1–56.0)	37.5 (27.1–50.7)
	7 d-FR	86.4 (59.4–124.4)	93.7 (66.3–133.4)	74.9 (52.2–116.3)*
	Difference (%)	−54.6	−58.2	−49.9
Fruits and vegetables (P)	DAT	3.0 (2.0–4.0)	3.0 (2.0–4.0)	2.5 (2.0–3.0)
	7 d-FR	2.0 (1.3–3.3)	2.0 (1.1–3.3)	2.1 (1.4–3.5)
	Difference (%)	50.0	50.0	19.0

All values are displayed as Median (Interquartile Range) in kilocalories (kcal), grams (g), or portions (P) per day. Difference in percent is calculated as follows: (DAT/7 d-FR)*100.

*DAT, dietary assessment tool; 7 d-FR, Seven-day food record.*

*Different from age group 20–40 years (p < 0.05).

**Different from age group 20–40 years (p < 0.01).

**FIGURE 1:**
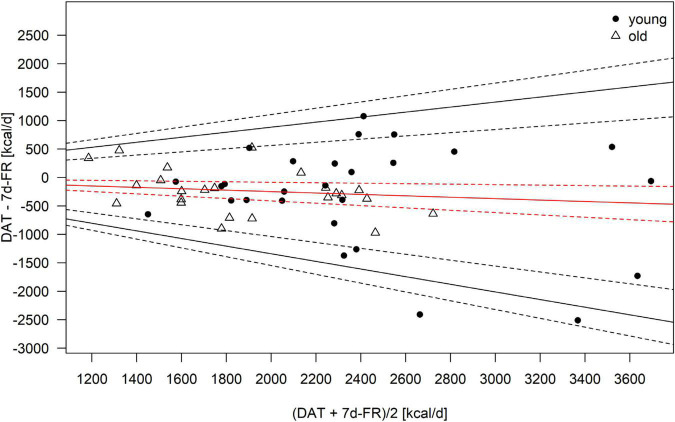
Bland–Altman Plot of the total calorie intake as calculated from the 7-day food record (7 d-FR) and the visually aided dietary assessment tool (DAT). Legend: Red solid line, mean; black solid lines, 95% limits of agreement; dotted lines, 95% of the respective solid lines.

**FIGURE 2:**
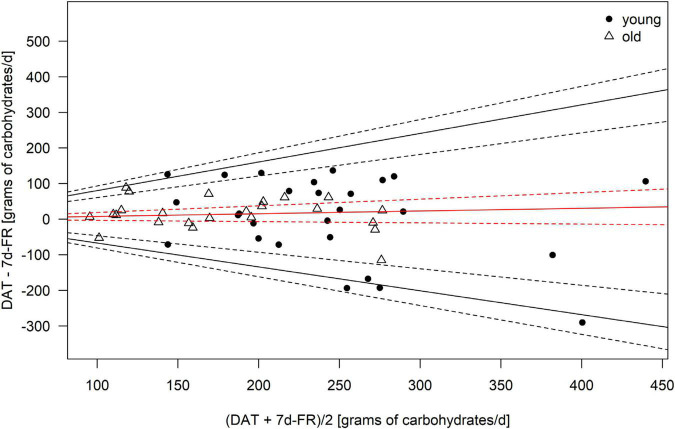
Bland–Altman Plot of the carbohydrate intake as calculated from the 7 d-FR and the visually aided DAT. Legend: Red solid line, mean; black solid lines, 95% limits of agreement; dotted lines, 95% of the respective solid lines.

**FIGURE 3:**
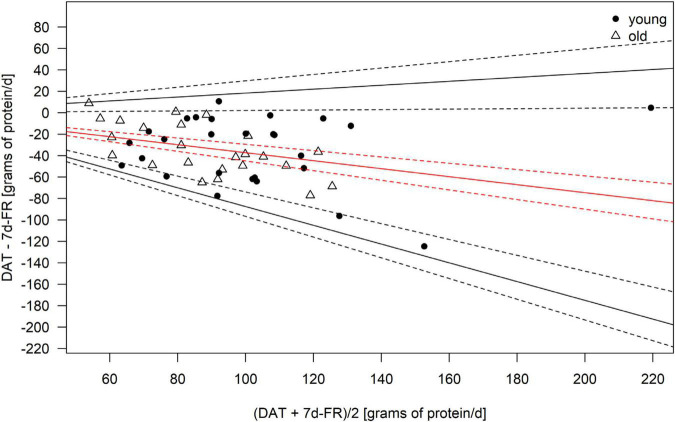
Bland–Altman Plot of the protein intake as calculated from the 7 d-FR and the visually aided DAT. Legend: Red solid line, mean; black solid lines, 95% limits of agreement; dotted lines, 95% of the respective solid lines.

**FIGURE 4:**
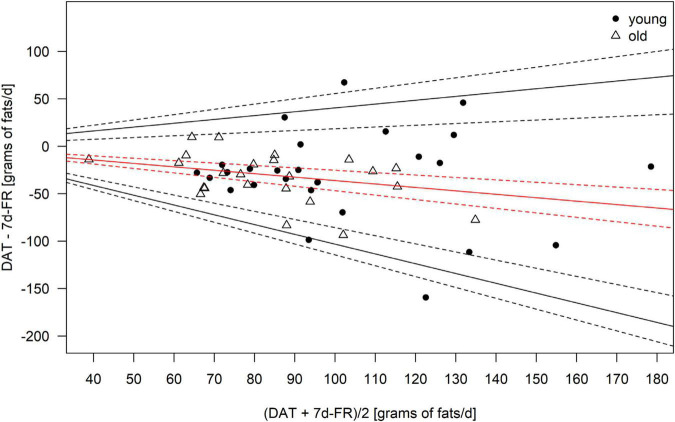
Bland–Altman Plot of the fat intake as calculated from the 7 d-FR and the visually aided DAT. Legend: Red solid line, mean; black solid lines, 95% limits of agreement; dotted lines, 95% of the respective solid lines.

Because the data was not normally distributed, all macronutrients, as well as total calorie intake, were logarithmically transformed (log10) for the analyses. Bland–Altman plots (Figures 1–4) were created for the log-transformed variables and transformed back to the original scale, as suggested by Euser et al. (31). The 95% limits of agreement for the Bland–Altman plots were calculated as the average difference ± 1.96 standard deviations of the difference (32). In accordance with Gerke (33), we created QQ-plots, histograms of the differences, and histograms of the results of the Preiss-Fisher procedure, which were all non-problematic (not reported) (33). The Bland–Altman plots were created for the entire population and not by age group, as the *p*-values of the log-transformed data of DAT – 7 d-FR were significant for both age groups. The correlation between macronutrients and water intake between the DAT and the 7 d-FR were calculated using Pearson’s r (Table 2). To check for any abnormal weight changes, a paired-samples *t*-Test was run between groups for pre and post measurements. Statistical analyses were performed using SPSS (IBM SPSS version 27.0. Armonk, NY, United States). All tests were performed two-sided and *p*-values < 0.05 were considered significant.

**TABLE 2:** Correlations between the DAT and 7 d-FR, by group.

Group	Kcal	Protein	Carbohydrates	Fats	Sugar	Water
Total	0.468	0.596**	0.483**	0.292**	0.288*	0.729**
20–40 years	0.277	0.584**	0.228	0.136	0.184	0.728**
50–70 years	0.799**	0.606**	0.776**	0.494*	0.479*	0.650**

*Significant correlations (p < 0.05).

**Significant correlations (p < 0.01).

*DAT, dietary assessment tool; 7 d-FR, seven-day food record.*

## Results

One subject did not follow the study protocol correctly and was excluded from the analyses; hence, 51 subjects were included. Participant characteristics are depicted in Table 3. The age range was 21–67 years, with an average age of 24.3 years in the young group and 57.4 years in the old group. There was a significant difference between the groups regarding height, body mass index (BMI), waist-to-hip ratio (WHR), and diastolic blood pressure. No statistically significant difference was observed for systolic blood pressure; however, it has to be noted that three participants of the older group were taking blood pressure-lowering medication. In addition, there was no significant weight change between the first and second visits in either group.

**TABLE 3:** Characteristics of participants by age group.

	Total	20–40 years	50–70 years
Female, n (%)	51 (47.1)	27 (40.7)	24 (54.2)
Age (*years*)	39.9 (17.1)	24.3 (2.7)	57.4 (4.5)**
Height (*cm*)	174.6 (9.3)	177.4 (10.2)	171.5 (7.2)*
Weight pre (*kg*)	72.0 (13.8)	71.2 (14.8)	73.0 (13.0)
Weight post (*kg*)	71.8 (13.7)	70.9 (14.7)	72.8 (12.8)
LBM pre (*kg*)	30.9 (1.0)	32.8 (1.5)	28.9 (5.7)**
LBM post (*kg*)	30.9 (1.0)	32.6 (1.5)	28.9 (5.8)**
Fat mass pre (*kg*)	17.0 (9.2)	12.8 (8.1)	21.7 (8.0)**
Fat mass post (*kg*)	17.0 (9.3)	13.2 (8.2)	21.2 (8.8)**
BMI (*kg/m*^2^)	23.5 (3.4)	22.4 (3.1)	24.7 (3.3)*

*Data are mean (SD) unless stated otherwise.*

*Different from age group 20–40 years (p < 0.05).

**Different from age group 20–40 years (p < 0.01).

*BMI, body mass index; LBM, lean body mass.*

We found significant differences between the 20–40 and 50–70 groups with regard to total energy intake (*p* < 0.01) and grams of carbohydrates (*p* = 0.03) for the DAT and 7 d-FR (Table 1). Further, we found that subjects aged 50–70 estimated significantly lower sugar consumption using the 7 d-FR than subjects aged 20–40. The results of the present study (Table 2) display that this DAT shows a high correlation with the reference method in the older group. The highest correlation was found for total energy intake in the older group at 0.799, which was much higher than the young group (0.277, *p* < 0.01). However, although the correlation for total energy intake in the old group was higher than the young group, the mean difference between total energy intake in the young group was lower (13.5%) than the old group (14.7%). In addition to total energy intake, correlations between the DAT and the 7 d-FR were significantly higher in the old versus the young group for carbohydrates (0.776, vs. 0.228, *p* < 0.01), fats (0.494, *p* < 0.05 vs. 0.136, *p* < 0.01), and sugar (0.479, *p* < 0.05 vs. 0.184, *p* < 0.01). No significant differences were observed for the other variables.

Regarding weight changes, we found that both groups were lighter at the second visit (0.3 kilograms in the young group, *p* = 0.06 and 0.2 kilograms in the old group, *p* = 0.04), with weight changes ranging from +1.7 to −2.1 kilograms. Only the weight change in the old group was significant (*p* = 0.04). No significant changes in lean body mass were seen in either group during the study (all *p* ≥ 0.2). We found changes in fat mass in the young group (*p* < 0.01) but not the old group (*p* = 0.375). Table 4 shows the mean bias, as well as upper and lower limits of agreement for the Bland–Altman plots. Due to the data being back transformed on the original scale, Table 4 further shows the mean slopes, as well as the slopes for the upper and lower 95% limits of agreement for total energy intake and macronutrients.

**TABLE 4:** Mean bias and limits of agreement (LoA) of the Bland–Altman plots for total energy intake and macronutrients.

Variable	Mean bias	LoA upper CI	LoA lower CI	Mean bias slope	LoA upper CI slope	LoA lower CI slope
Kcal	0.054	0.303	−0.195	−0.124	0.442	−0.671
Carbohydrates	−0.033	0.303	−0.369	0.077	0.803	−0.671
Protein	0.164	0.408	−0.080	−0.373	0.183	−0.875
Fats	0.159	0.496	−0.178	−0.362	0.404	−1.032

*Legend: CI, 95% confidence intervals.*

The Bland–Altman plots are shown in Figures 1–4. Mean and 95% limits of agreement are displayed as solid lines, with the respective 95% confidence intervals displayed as dotted lines. As visible in and Figures 1–4 and Table 1, the DAT appeared to overestimate total calorie intake, protein, and fat intake, whereas carbohydrates intake was underestimated. Bland–Altman analyses were not possible for fruit and vegetables intake, as they did not fulfill the necessary statistical criteria. In addition, all subjects in the old group were within the limits of agreement for all parameters. The difference between DAT and 7 d-FR was 237 kcal for total energy intake, 35.1 grams (144 kcal) for protein, 76.7 grams (314 kcal) for carbohydrates, 32.8 grams (295 kcal) for fats, and 47.2 grams (194 kcal) for sugar (Table 1).

## Discussion

The present study aimed to assess whether the visually aided dietary assessment tool (DAT) is a valid instrument to estimate food intake in Swiss adults, aged 20–40 and 50–70 years. The gold standard 7 d-FR was used as a reference method and validity was investigated for all macronutrients (in grams), as well as total calorie consumption (in kilocalories), amount of portions of fruits and vegetables, and sugar intake (in grams). The age groups were defined in a manner to discriminate between young adults, who tend to have less regulated daily routines than older adults (23, 34). As demonstrated by Willet (3), FFQs are commonly used to assess long-term dietary habits, which was the key area of interest in the present study (3). We showed that the young group was able to estimate energy intake and all macronutrients more precisely than the old group. Across both groups, total energy intake, carbohydrates, and portions of fruits and vegetables were estimated more precisely than protein, fats, and sugar. However, correlations between the DAT and 7 d-FR were stronger in the old group, with significant correlations for total energy, protein, carbohydrates, fats, sugar, and water. The only significant correlations that were observed in the young group were for protein and water. Notably, the correlation of 0.799 for total energy intake in our old group was much higher than in comparable, recent studies (35, 36).

Our study reveals that the DAT we used overestimated total energy intake. This is in line with other European studies, that reported differences between the DAT and FR (37, 38). However, the recent systematic review by Sierra-Ruelas et al. (39) revealed that most DATs tend to underestimate total energy consumption. While the overestimation of energy in the overall population is acceptable (+12.3%), the Bland–Altman plots revealed that the DAT mostly overestimated fat and protein intakes, and that carbohydrates were underestimated. Since fats have an energy density of 9 kcal/g, an overestimation of fat consumption will inevitably affect the estimation of total energy intake. A potential explanation for the overestimation of fat intake in the DAT might be, that fats are highly represented in the DAT, making up a fast part of the upper section of the DAT. Further, the portion size of high-fat food items such as cheese or nuts is relatively high in the DAT, creating an overestimation of the consumption of high-fat foods. To our knowledge, we are the first to report back-transformed data in Bland–Altman plots for the validation of a DAT or FFQ. Therefore, we cannot precisely compare our results with other data. However, when comparing our findings with a similar validation study undertaken in Switzerland (28), we hereby show that the DAT we used appears to be more precise, especially in the older population which consumed less calories. However, no comparison can be made for the macronutrients.

Regarding the assessment of water consumption, there is no consensus on how to precisely assess intake (40). However, as stated in an overview by Mons et al. (41), food records should be preferred over retrospective methods because of higher precision. In our study, we were able to demonstrate that contrary to the current consensus, our method assessed water intake with high precision. In contrast, our study showed that sugar intake was poorly estimated with a correlation between the DAT and 7 d-FR of 0.184 (*p* = 0.40) and 0.479 (*p* < 0.05) in the young and old groups, respectively. Our population in the young group estimated a daily sugar intake of 50.6 (± 30.8) grams, which would be in accordance with the WHO recommendations. The old group estimated their sugar intake at 39.3 (±17.1) grams per day. However, the 7 d-FR showed that daily sugar intake was approximately twice as high (101.7 grams in the young and 81.6 grams in the old group) in both groups, indicating that sugar intake estimation was a challenge regardless of age. The intake measured in the 7 d-FR is in line with results published in 2019 on sugar consumption in Swiss adults (18). The authors found that in a population aged 18–75 years of age total daily sugar intake equaled 107 grams on average, which is comparable to our results of 92.2 grams (101.7 grams for the young group, 81.6 for the young group).

We found that both groups lost some weight during the study, on average 0.2 kilograms. It has been well documented that study subjects may change their dietary patterns to a healthier approach when being monitored to fit social desirability (7). However, as recently reported by Turicchi et al. (42), within-week weight fluctuations of up to 0.35% in body weight can be observed. This placed both of our groups within acceptable weight change ranges.

The present study has several limitations. Firstly, the assessment method using a visual aid with a colored pyramid is suggestive. Study subjects see which food items are more socially accepted, as they belong to the food category which has a “healthy” image. These categories are shown in green or blue colors, in contrast to yellow, brown, or red for food items that are regarded as less desirable. However, the food pyramid is well known in Switzerland and therefore all participants were familiar with it.

The portion size equivalents shown in the food pyramid and estimated by the SGE were relatively broad. For example, a portion of bread ranged from 75 to 125 grams, and the type of bread is not specified. Since older people generally consume smaller portions than younger adults, this may have led to an overestimation of the portion size in the old group, and an underestimation in the young group (34). Portion size estimation is considered one of the main reasons for inaccurate reporting in food questionnaires, and different portion sizes for various aged groups may be a valid solution (43).

Further, study personnel was present when participants filled out the DAT. Although study personnel was not actively watching the participants but rather performing other tasks, the presence of the personnel may have affected the estimations of the participants. In addition, the keyword “typical day” when assessing dietary habits was not defined using a specific period (e.g., last seven days, last month, or last year). While dietary intake may vary because of seasonality in some populations, an analysis using data from Swiss studies determined that seasonality decreased in the last decades and may not play a significant role today (44). A limitation of our analyses is that we did not define limits of agreement *a priori* based on biologically and analytically relevant criteria, as has been suggested for the Bland–Altman plot system by Giavarina (45). However, we are not aware of any validation studies with comparable dietary assessment methods (e.g., food frequency questionnaires), where the suggested approach has been applied and reported. Lastly, we determined our sample size according to previous studies but did not calculate the power for our analyses, therefore potentially not recruiting enough participants.

All participants used their private household scales since a standardized kitchen scale was not provided. No calibration of scales was therefore available. This may have led to discrepancies when weighing the food items and to systematic over-or underestimation of portion size. Therefore, energy intake as measured with the 7 d-FR can only be estimated.

We also did not monitor physical activity. Weight loss, as observed in our study, is the result of negative caloric balance. This is achieved either by lowering the total energy consumption or increasing physical activity and therefore energy requirements. It is possible that the old group did not achieve the weight loss through nutritional changes but rather by increasing physical activity. Lastly, the food pyramid that was used for the COmPLETE study and the present validation study was in use between 2005 and 2011. While there were no major changes in the pyramid since then, some trendy food (e.g., tofu, nuts, beans, lentils) may be underrepresented. The main strength of the present study was the use of the 7 d-FR, which is regarded as superior to retrospective assessment methods for this kind of validation study. In addition, most, if not all meals were consumed at home due to the restrictions of the COVID-19 pandemic. This may have led to more precise dietary monitoring during the study.

## Conclusion

The present study showed that a simple dietary assessment tool can be used effectively in an adult Swiss population. The highest correlation between the DAT and the gold standard 7 d-FR was achieved in people aged 50–70 years old but younger, as well as older adults overestimated total energy, protein, fats, and fruits/vegetables portions. Sugar intake was strongly underestimated. To conclude, this DAT appears to be a valid alternative to the more complex weighed food records in epidemiological studies to estimate dietary habits but not to calculate precise macronutrients intake.

## Data Availability Statement

The raw data supporting the conclusions of this article will be made available by the authors, without undue reservation.

## Ethics Statement

The studies involving human participants were reviewed and approved by the Swiss National Science Foundation (SNSF) grant no. 182815. The patients/participants provided their written informed consent to participate in this study.

## Author Contributions

AS-T and GN were responsible for conceptualization and methodology. GN, LB, and LL were in charge of recruitment and conducting the research. GN performed the statistical analyses, and wrote the manuscript. CH, CB, and NS provided expert advice for statistical analysis and manuscript review. AS-T was responsible for funding acquisition. All authors read and approved the final manuscript.

## Conflict of Interest

The authors declare that the research was conducted in the absence of any commercial or financial relationships that could be construed as a potential conflict of interest.

## Publisher’s Note

All claims expressed in this article are solely those of the authors and do not necessarily represent those of their affiliated organizations, or those of the publisher, the editors and the reviewers. Any product that may be evaluated in this article, or claim that may be made by its manufacturer, is not guaranteed or endorsed by the publisher.
