# Supplementary material for: Validation of a Visually Aided Dietary Assessment Tool to Estimate Dietary Intake in an Adult Swiss Population
Source: Front Nutr. 2022 Apr 28;9:844156. doi: 10.3389/fnut.2022.844156 (PMC9097151; doi:10.3389/fnut.2022.844156)
Supplement: Supplementary file 1 [file Data_Sheet_1.PDF]

Visite 1 / 2

FFQ 1 ____________

Ernährungsprofil		Portionen/Tag					
	**1 Portion entspricht entweder/oder**	**Frühstück**	**Zwischen-mahlzeit**	**Mittag-essen**	**Zwischen-mahlzeit**	**Abend-essen**	**Portionen**
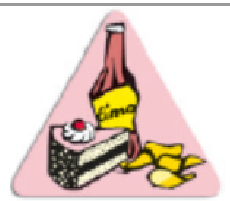	25 g Schokolade (1 Reihe/Riegel)<br>3 dl Süssgetränk<br>25–30 g salzige Snacks<br>3–4 Kekse (Guetzli)<br>30 g Kuchen (1 kleines Stück)						Portionen/Tag
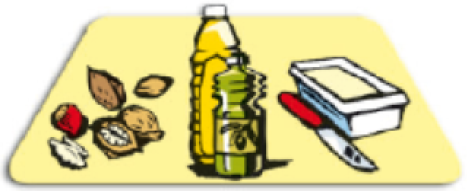	10 g (1 EL) hochwertige Pflanzenöle (wie Raps-, Baumnuss-, Olivenöl) für die Zubereitung<br>10 g Butter oder Margarine<br>20–30 g (1–2 EL) ungesalzene Nüsse						Portionen/Tag
	fettreiche Speisen:<br>Frittiertes, Paniertes, Rahmsaucen, ölhaltige Saucen, Wurstwaren (50–100 g)						Portionen/Woche
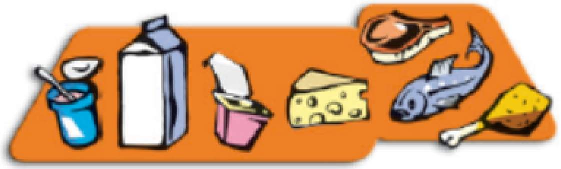	100–120 g Fleisch/Geflügel/Fisch<br>100–120 g Tofu, Quorn<br>2 Eier						Portionen/Tag
	2 dl Milch<br>180 g Joghurt/Sauermilch<br>150–200 g Quark/Hüttenkäse<br>40 g Hartkäse<br>60 g Weichkäse						Portionen/Tag
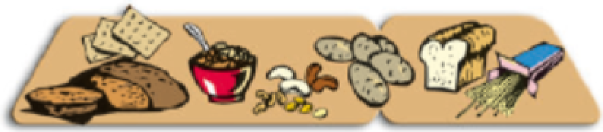	75–125 g Brot<br>45–75 g Getreideflocken, Teigwaren, Reis, Mais oder anderes Getreide (Rohgewicht)<br>180–300 g Kartoffeln<br>30–40 g Frühstücksflocken<br>60–100 g Hülsenfrüchte (z. B. Linsen)						Portionen/Tag
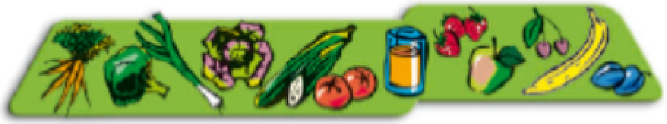	Mind. 120 g Gemüse/Früchte (1 Handvoll)<br>2 dl Gemüse-/Fruchtsaft<br>50 g Blattsalat						Portionen/Tag
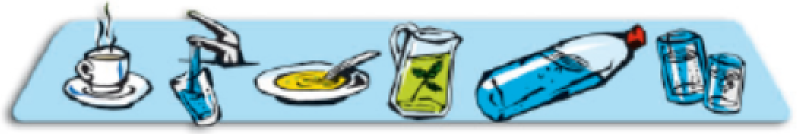	Ungesüsste Getränke<br>(Wasser, Mineralwasser, Tee, Kaffee)<br>1 Glas/1 Tasse (1.8–2 dl)						Portionen/Tag
	Alkoholische Getränke<br>Standarddrinks (nicht für Jugendliche)<br>1 dl Wein oder 3 dl Bier						Portionen/Tag
Quelle: Schweizerische Gesellschaft für Ernährung SGE							
